# Experiencing art together: integrating affect and semiosis

**DOI:** 10.3389/fpsyg.2025.1544901

**Published:** 2025-04-30

**Authors:** Gemma Schino, Lisa-Maria van Klaveren, Theisje van Dorsten, Samrddhee Pathare, Barend van Heusden, Ralf F. A. Cox

**Affiliations:** ^1^Department of Psychology, University of Groningen, Groningen, Netherlands; ^2^Institute for Education and Training, Amsterdam University Medical Center, Amsterdam Public Health Research Institute, Amsterdam, Netherlands; ^3^University College Groningen, University of Groningen, Groningen, Netherlands; ^4^Department of Arts, Culture, and Media, University of Groningen, Groningen, Netherlands

**Keywords:** art experience, affect, semiosis, sentiment analysis, exploratory graph analysis

## Abstract

**Introduction:**

Art is ubiquitous in our lives, and its experience and understanding are deeply emotional. Dewey suggested that all human experience, including art experiences, emerges from active engagement with the environment. In this view, affect and interpretation are interconnected processes that unfold together. To examine the integration of these processes, this interdisciplinary study used a multi-method approach.

**Methods:**

Eighteen dyads of adult participants took part in the study. They were instructed to each bring an art object that was meaningful to them. During the experiment participants engaged in an audio-visually recorded, semi-structured conversation, reflecting on both art objects. They also answered pre- and post-questionnaires on their emotions. Affect was measured through self-reported valence and arousal of emotions, and sentiment analysis of the conversation. Semiosis as the process of making sense of the art objects was operationalized in terms of four strategies, namely: perception, imagination, conceptualization, and analysis. Affect was measured through self-reported valence and arousal of emotions, and sentiment analysis of the conversation.

**Results and discussion:**

The results showed that dyadic interactions led to changes, at the group level, in participants’ self-reported affect toward the other’s art object. An Exploratory Graph Analysis revealed unique weighted networks of sentiment for each strategy. Additionally, a Multinomial Log-linear Model demonstrated that affect and strategies work in tandem during the art experience, to predict perceived affect.

## Introduction

The human capacity to appreciate art is seemingly universal (Dutton et al., 2009; Wah, 2017): always, and everywhere, people have reached out to art to come to grips with experience (Donald, 2006). Art comes in many forms, such as performing arts, visual arts, design and craft, literature, online and digital arts. We encounter it in community- and cultural festivals, museums and galleries, on the streets and fairs, and in events that intersect with art practices (Davies et al., 2012). According to Dewey (1934), the experience of art results from an interaction with the environment. Art experiences are, therefore, deeply rooted in the particulars of human cognition. While art is often thought of as an observable, empirical quality of certain objects or events –“works” of art like paintings, sculptures, music, or performances– it may be more accurately described as a process, an activity that human beings can undertake with these objects. As van Heusden (van Heusden, 2015; Noë, 2016) suggests, the essence of art lies not in the art object but in the act of sense-making it incites. This process entails evaluative and non-indifferent responses (Colombetti, 2014) linking individual personal experiences with larger structures, such as social, contextual, institutional, and historical elements (McCallum et al., 2020). Thus, an art experience is best understood, not as a static property of (art) objects, but as an active, dynamic process. This process involves both *affect* (including behaviors, physiological changes, mood, sentiment, and emotions) and *semiosis* (making sense of the art objects through a variety of semiotic strategies). The present study aims to explore the intricate interplay between affect and semiosis in art experiences. We will first briefly introduce the three main concepts, i.e., art experiences, affect and semiosis, to then bring them together in the proposed model and study.

Philosophers such as Kant, Croce, and Bell have explored the receptive experience of art, and aesthetic judgements, laying the groundwork for empirical investigations in this domain (Leder et al., 2004). Researchers from different disciplines have been interested in researching art and the experience it brings, which is often infused with diverse emotions (Scherer, 2004; Silvia, 2005b; Menninghaus et al., 2019). The theory of aesthetic cognitivism conceptualizes art experiences as a form of knowledge (Graham, 1997; Gaut, 2003; Baumberger, 2013; Christensen et al., 2023a). While the aesthetic can elicit experiences of beauty, the arts are more than sources of delight, amusement, or pleasure (though they can certainly be all of these). Art does not need to entertain: the value of the arts is in them being a means of reflection. From a cognitive perspective, aesthetics is viewed as a branch of cognitive science that focuses on the psychological mechanisms underlying aesthetic experiences (Wassiliwizky and Menninghaus, 2021). Wassiliwizky and Menninghaus (2021) argue that empirical aesthetics has made its way into mainstream cognitive science, and advocate for more research in the temporal dynamics and interaction between the art object and the perceiver, as well as different systems within the perceiver (Cox et al., 2023).

Previous research into the complexity of multiple interconnected factors implicated in art experiences has shown that our engagement with paintings, literature, and/or music is essentially accompanied and informed by emotions (Schindler et al., 2017). Art evokes a myriad of responses, a variety of subjective thoughts and feelings (Schindler et al., 2017), evaluations (Leder et al., 2004), physiological reactions (Tröndle and Tschacher, 2012), and behaviors (Dissanayake, 2008). These responses play a role in the experience of various art forms, including paintings (Leder and Nadal, 2014), music (Scherer and Coutinho, 2013), literature (Mar et al., 2011), film, and television (Bartsch and Oliver, 2011). To bring together the different types of responses under a comprehensive conceptual umbrella, we draw from the Latin notion of *affectus* (which refers to affection, mood, emotion, feeling, disposition, condition or state of body or mind all at the same time). Affect can then be defined as the lack of indifference. By affect we mean physical and mental states characterized by valence (pleasant or unpleasant feelings), arousal (low or high activation), and intensity (strength of the feeling) (Barrett, 2006; Lindquist, 2013). Affect encompasses behaviors, bodily (physiological) changes and subjective feelings. Zooming in on the latter, these feelings include, but are not limited to, sentiment (as the attitude toward the event or experience) and perceived affect, such as emotions with different levels of valence, arousal, intensity and often labeled in terms of categories (like happiness, fear, and so on; Cupchik, 2016). Frijda (2007) argues that emotions have an adaptive function guiding behavior in the physical and social world.

The last two decades have witnessed numerous empirical and theoretical attempts at understanding the complex connection between art and affect (Skov and Nadal, 2020; for an overview, see Nadal and Vartanian, 2022). While some research supports the idea that appreciation and engagement with art are fully detached and devoid of emotions (James, 1890; Carroll, 2012). Other research, however, contends that affect plays a determinant role in such experiences (Cooper and Silvia, 2009; Prinz, 2011; Pelowski et al., 2016): artworks move us causing various emotions, such as wonder (Fingerhut and Prinz, 2020), and art experiences are therefore non-indifferent. As such, they should not be treated as a separate phenomenological class of emotions and shall be defined to be “multi-componential, including subjective feeling, appraisals, reactions in the service of action preparation and expressions, action tendencies (including expressions), and regulation” (Sacharin et al., 2012, p. 1). Different factors and components interplay in this process, to the point that the experience of art involves bodily feelings and behavioral responses (Freedberg and Gallese, 2007; Colombetti and Krueger, 2015). Building on this perspective, we argue that art-elicited affect is not necessarily different from affect experienced elsewhere: such responses in art likely function within the same broad processes as they do in other contexts. This may be especially pronounced in cases where an artwork holds personal significance –our response cannot be neutral but must be embodied, as we engage with it through lived experience. We experience and feel them in our self, as a lived body, through incorporation (Merleau-Ponty, 1945). As Colombetti (2016; 2017) proposes affective responses to art are not limited to intense emotions but can also involve subtler, non-emotional modes of engagement. This suggests that our responses to art do not have to be intensely emotional to be considered affective or meaningful.

This phenomenological view aligns with Dewey’s theory of emotions (Dewey, 1895). In art experiences, we may experience bodily changes and shifts in mood without them being intense but, when intense emotional reactions do occur, they often arise as part of a reflective process. In Dewey’s words (Dewey, 1934, p. 15): “Emotion is the conscious sign of a break, actual or impending. The discord is the occasion that induces reflection. Desire for restoration of the union converts mere emotion into interest in objects as conditions of realization and harmony”. In this sense, reflection is a form of sense-making, a process of engaging with the difference or disruption in experience to restore balance and meaning. For instance, appraising something as dangerous and feeling fear are not distinct experiences but rather two aspects of the same experience. Damasio’s somatic marker hypothesis (Damasio, 1999) further supports this, suggesting that bodily states –or “somatic markers”– link physiological responses with conscious interpretations (i.e., the emotion perceived and categorized). Damasio (1994) argues that emotions shape and enrich human thinking, just as reflecting, understood as sense-making, in turn informs and tempers our affective responses.

The role of emotions in art experiences has been explained in diverse ways in current psychological models of art. These explanations range from visual object identification (Chatterjee et al., 2010), intellectual art expertise (Leder and Nadal, 2014), to relative matching of schema and self (Silvia, 2005b). The Vienna Model of Art Perception (VIMAP, for more information see Pelowski et al., 2017) integrates these perspectives, providing a comprehensive framework that encompasses visual identification, intellectual engagement, personal relevance, and analytic approaches to explain the complex process of emotional and evaluative responses to art. However, the models outlined so far often tend to overlook the role of semiosis in their frameworks.

Following Peirce (1932) and Kull (2022), *semiosis* can be described as a triadic process “in which the field of possibilities (firstness) is interpreted (secondness) into something modified (thirdness)” (p. 7). Semiosis, or sense-making, is an action involving the choice and realization of one possibility. Van Heusden (2009) argues that “sense-making with art” facilitates reflection.

In our framework, semiosis is operationalized through the employment of four cumulative semiotic strategies while we intentionally engage with our personal, social, and natural spheres: perception, imagination, conceptualization and analysis (Van Heusden, 2009). *Perception* relates to the sensory (aesthetic) experience of physical properties such as touching, seeing, smelling, etc. Arnheim (1954) set forth Gestalt principles to understand how balance, symmetry, and composition create different kinds of aesthetic experiences (Chatterjee and Vartanian, 2014; van Geert and Wagemans, 2020). The perceptual is one of the building blocks on which other semiotic strategies can develop. Going forward, *imagination* entails the power of forming, retaining, and manipulating mental images/schemas (Wah, 2017). Imagination also allows humans to deal –reflexively– with their own experiences via “self-imagination” –a strategy that involves the recreation of one’s or others’ experiences (van Heusden, 2004; Wah, 2017). Therefore, imagination, if combined with reflection, is the cognitive basis for art, allowing one to build upon perception, making art experiences highly individual. For these reasons, imagination grounds human creativity, underpinning all human creations, from simple tools to complex instruments. *Conceptualization* allows for the categorization, classification, and valuation using concepts conveyed through language. This strategy helps interpreting and categorizing art by drawing on definitions, similarities, or prior knowledge (van Heusden, 2015; Seeley, 2020). In doing so, it directs perception and guides our understanding of art’s purpose. *Analysis* builds on perception, imagination and conceptualization. This strategy makes sense of the experience in terms of structures that are discovered through observation (van Heusden, 2015; van Klaveren et al., 2023). Importantly, semiotic strategies often occur in parallel or sequentially, and develop over time (van Dorsten, 2015).

**FIGURE 1:**
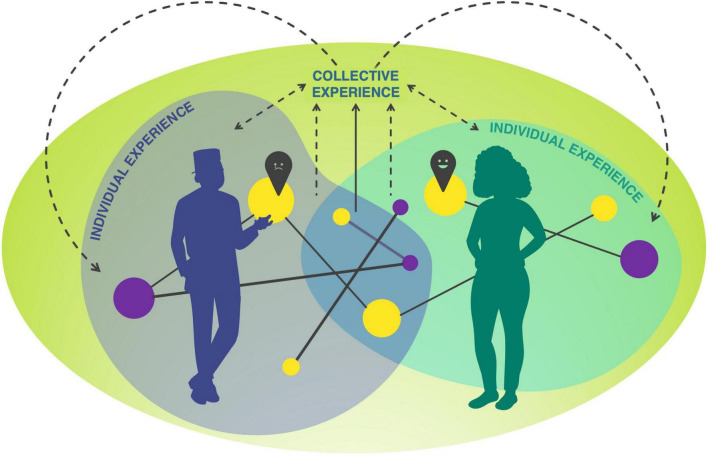
The proposed model. Art experience can occur at both individual and collective levels, incorporating affective elements (respectively, sentiment represented as yellow circles and perceived affect as pins) and semiotic strategies (represented as violet circles) that interact dynamically (shown as lines). The model includes “checkpoints” –moments within the process that occur above our threshold of awareness and manifest as perceived affect (represented as pins). This interaction of affective aspects and semiotic strategies shapes the experience itself, creating a feedback loop that informs and refines future experiences.

Art objects serve as a medium through which individuals can reflect not only upon their own lives, experiences, and emotions but also on those of others, as well as on broader aspects of life and existence itself (Donald, 2006). We conceptualize the art experience as a process that encompasses, on top of its core of imaginative reflection, perceiving, thinking, feeling, and much more. To encapsulate this integration, a new model (see Figure 1) is proposed. This model illustrates the dynamic, iterative process through which individuals experience and make sense through art at the individual and collective levels, including both affect and semiotic strategies. Although we acknowledge that many more aspects of affect, and, more broadly, cognition –such as bodily sensations, behavioral changes, and physical responses– also play roles in shaping the art experience, this study will focus on affective aspects (specifically, sentiment and perceived affect) and semiotic strategies employed during the art experiences. An experience becomes collective when two or more individuals engage, usually in dialogue, to negotiate and integrate their perspectives, interpretations, or insights. “By turning it [the subjective experience] into a collectively shareable object, language allows it to be incorporated, redefined and reshaped in different contexts and world views” (Ferreira, 2015, p. 1135). Discussing through speech and discourse allows people to reflect on the experience and co-construct meaning (Barthes, 1977; Cassirer, 1985; Percy, 1995). Language does more than articulate experience –it actively structures it (Brooks et al., 2016). Besides being a window on our thoughts and cognition (Tenbrink, 2015), language facilitates the acquisition of new concepts and shapes sensations (Lupyan et al., 2007; Lupyan, 2012a,b). In this sense, the interaction between individuals becomes a way of building upon an already reflective experience (the art experience, Cupchik, 1995; Norman, 2004), allowing individuals to deepen their experience on top of previous ones. This iterative, collaborative and reflective process not only influences how the artwork is experienced but also transforms the experience itself by incorporating new information.

To sum up, with this model, we posit that affect and semiotic strategies during art experiences are deeply intertwined and mutually influential. Emotional responses should not be considered as “endpoints” (i.e., products of a process) following from art experiences. Art experiences are not a static precursor to emotional reaction; rather, affect and semiosis work in tandem, co-constructing the perception and overall experience of the artwork (see Vartanian et al., 2024): trying to pinpoint the nature of this interaction is the focus of this research.

The present study aims at exploring the integration of affect and semiotic strategies in individual and collective art experiences. To this end, we have designed a study that seeks to mimic the natural context in which these experiences often occur.

Firstly, contrary to the conventional view that art experiences are confined to museums or theaters, they can, in fact, happen anywhere “in the wild” –from scrolling through social media at home to streaming a film online (Huang and Su, 2018; Christov-Moore et al., 2024). Secondly, it is important to distinguish between the experience of art and the reflection on and with art. The latter can so often be an integral part of the experience itself; for example, we frequently share our impressions with others after watching a movie (reflecting *on* art), thereby shaping the meaning of the experience through dialog and social discourse (Ferreira, 2015) and ultimately drawing connections between the art and our own lives, and the existence in general (reflecting with art). Therefore, we designed a quasi-experiment that would aim at reproducing this type of experience in a somewhat naturalistic manner and investigate the reflection on top of an a priori reflective experience, which is the art one. This research makes use of an interdisciplinary and multi-method approach (Schino et al., 2022). We invited adults to participate in an in-lab experiment together with a friend, peer, partner, or family member. Each pair of participants came to the lab bringing two art objects (i.e., artworks and/or objects artfully crafted; one per participant) that were meaningful to them. To self-report their reactions, they each answered questionnaires about the art objects before and after engaging in a video-recorded dyadic interaction to stimulate a collective art experience and to use speech as a window of participants’ thoughts (Tenbrink, 2015). The data provided us with the following information:

1. Affective aspects: we delve into the subjective feelings involved in art experiences. Affect is defined as: (1) sentiment, captured through text-mining of the transcripts from the dyadic interactions (Kenett et al., 2023); and (2) perceived aspects of affect, namely emotions and their valence, arousal, intensity, and specific categories, measured through the Geneva Emotion Wheel self-report tool (GEW) (Scherer et al., 2013) before and after the interaction.
2. Semiotic Strategies: we examine how participants make sense of art objects through a variety of strategies. These strategies –perception, imagination, conceptualization, and analysis– are identified and coded from the transcripts of participants’ interactions.

The study’s objectives are threefold: firstly, to examine changes in perceived aspects of affect (valence, arousal, intensity and categories of emotions) before and after the dyadic interactions; secondly, to investigate the relationship between affect and semiosis, employing computational sentiment analysis, alongside qualitative coding of semiotic strategies to uncover underlying connections; thirdly, to explore how these dimensions and their interactions influence the perceived aspects of affect in art experiences. Ultimately, this study takes an inductive approach to explore the overarching research question*: What is the interplay of affect and semiosis during art experiences?* And the sub-question*: Does the collective experience influence individually perceived affect?* More specifically, we are particularly interested in how affective aspects (perceived affect and sentiment) and semiotic strategies shape the art experience as they unfold and evolve through natural interactions.

This study is structured around three hypotheses, each progressively deepening the exploration: from the most straightforward examination of changes before-after and during the interaction, to a more intricate analysis of how the dynamics within the interaction shape the overall final affective outcomes of the experience.

*H1*: The dyadic interaction influences the perceived affect toward the art objects in terms of valence, arousal and intensity of emotions. *H2*: While we expect affect in terms of sentiment to be present in all semiotic strategies, we also hypothesize significant differences in sentiment between the specific strategies. *H3a*: Affective aspects of the art experience in terms of sentiment influence perceived affect post-dyadic interaction. *H3b*: Semiotic strategies influence perceived affect post-dyadic interaction. *H3c*: The interaction between affective aspects (sentiment) and semiotic strategies influences perceived affect post-dyadic interaction.

## Materials and methods

Both a quantitative and qualitative lens for data collection and analysis were employed. The purpose of this type of mixed research design was to encapsulate the experience of art in a comprehensive manner, especially since it is an inherently complex, intuitive, diverse process involving various meanings and interpretations (Starr and Smith, 2023). For privacy reasons, only an anonymized dataset may be available upon request for research purposes by emailing the corresponding author.

The study was approved by the Ethics Committee of the Faculty of Behavioral and Social Sciences at the University of Groningen (PSY-2223-S-0252) and was conducted according to the Dutch ethical standards for scientific research.

### Participants

This study consisted of 38 people (19 dyads) of 18 years or older (23 females, fifteen males, *M*_*age*_ = 24.18, *SD*_*age*_ = 7.59), who voluntarily participated in the study.^1^ The study took place from May 1st to May 19th, 2023. Participants were only screened for age (18+), while other demographic and cultural characteristics were not considered, as these variables were irrelevant to this study.

Recruitment of potential participants took place through convenience sampling. Recruitment methods included: (i) targeted advertisement via research panel website (SONA)^2^ aimed at first-year psychology students at the University of Groningen in the Netherlands; (ii) public advertisement on the communication/social media platforms (e.g., Facebook, Instagram, LinkedIn, Twitter, WhatsApp group chats); and (iii) flyer distribution at local centers for leisure, culture and educational activities. Participants could choose a type of compensation –SONA credits, a gift voucher worth €10 or a donation of €10 to schools for cultural activities.

### Procedure of data collection

**FIGURE 2:**
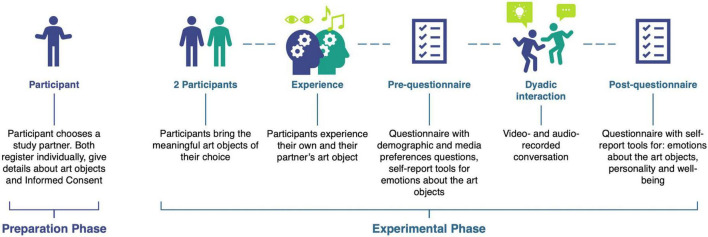
Overview of data collection process. During the Experimental Phase, ‘Experience’ refers to the participant’s engagement with the art objects through the allowed modalities (e.g.: listening, observing, touching, smelling, watching, etc.).

A multi-method approach was employed. Quantitative and qualitative data were gathered in multiple ways and in a naturalistic setting. Participants were instructed to bring a meaningful art object with them. They took part in the experiment as a dyad to observe their art objects and talk about them (herein described as “dyadic interaction”) and answered pre- and post-dyadic interaction questionnaires individually. The experiment was divided into two phases –the preparation phase and the experimental phase. Throughout the experiment, one researcher stayed in the room to instruct the participants and answer potential questions. Figure 2 illustrates the data collection process.

#### Preparation phase

Prior to the data collection, potential participants were given information about the study and were instructed to sign up with a known peer of their choice on mutual agreement. They were each asked to bring one meaningful art object such as a painting, photograph, film scene, song, favorite book or poem –created by artists, or even the participants themselves, in digital or physical form– to the experiment location (an overview of the type of art objects has been reported in Supplementary Figure 1A: Appendix A). They were instructed not to reveal their art objects to each other before the experiment, and to not bring anything that could potentially upset the other person. Art objects that could not be brought physically to the location were submitted digitally through an online form to the researchers in advance, for later use in the experiment. At this point, informed consent was obtained—digitally via email or on location via a Qualtrics^3^ survey.

#### Experimental phase

In this part of the experiment, each dyad took part in experiencing their and their peer’s art objects, filled out questionnaires and had a dyadic interaction to discuss the art objects. Upon arrival, each dyad was taken to a room to be seated together. A description of the lab setting is provided in Supplementary Appendix B. This was followed by “art appreciation” where each participant individually experienced their and their peer’s art object for a minimum of 20 s to a maximum of two-and-a-half min. The order of the objects was random. These experiences involved watching a film scene, observing a painting, listening to a song, or watching a video clip, depending on what was brought by the participants. After this, participants filled out a (pre) questionnaire via Qualtrics on a tablet regarding both their and their peer’s objects. This included various self-report tools,^4^ among them the Geneva Emotion Wheel (GEW) (Scherer et al., 2013) for emotional assessment of the experience. Upon completion, the art objects were swapped, and the process was repeated, starting from the art appreciation.

This was followed by the audio-visually recorded dyadic interaction where participants were instructed to stand up and converse about both the art objects they had just experienced. The rationale behind the design was to build a facilitating condition: “a setting that encourages open and reflective dialogue among participants” (Pizzolante et al., 2024, p. 12; see also Blackburn Miller, 2020; París and Hay, 2020). During the conversation, eight prompts were presented. They were used as guidance to keep the conversation flowing and ensure the strategies would be captured during the conversation (for more details, refer to the Measures section). Each prompt was displayed on a screen and was timed for 2-3 min. The entire interaction lasted a minimum of 10 min up to a maximum of 20 min to measure speech content. To keep track of time, a visual timer on a screen indicated the time participants had left to experience the object and discuss the prompt. Both art objects were placed on a table during the interaction, where they could be engaged with, if desired. The time of 20 min of having discussed all the prompts marked the end of the interaction. Then, the recordings were stopped, and the participants were asked to take a seat again. Next, the participants were instructed to fill out the (post) questionnaire (i.e., GEW).

### Measures

#### Perceived affect: Geneva Emotion Wheel

**FIGURE 3:**
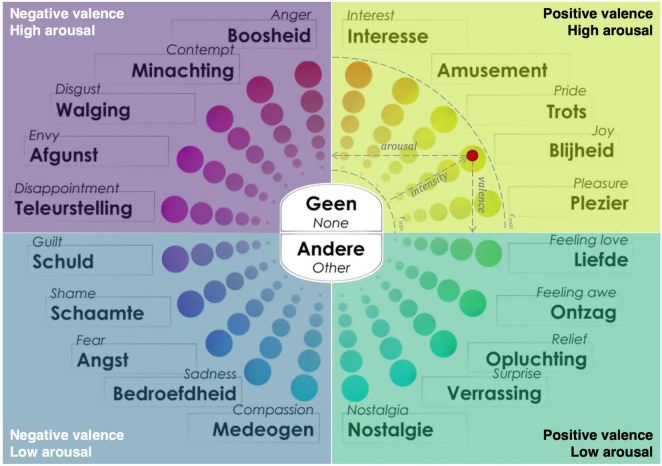
Dutch-English Geneva Emotion Wheel, its quadrants and information for calculations. The wheel was within a pixel grid of 900 × 632, and it has a diameter of 496 pixels, cantered at coordinates (465, 316). The red dot represents an example point at the measure that can be derived from its xy-coordinates. The wheel organizes emotions within a 2D space based on the circumflex model of emotion (Russell, 1980). This allows us to infer two dimensions: Valence (represented on the x-axis) and Arousal (represented on the y-axis). Clicks are positioned in specific quadrants within the wheel, making it possible to calculate these dimensions using the x and y coordinates of each selected emotion. For example, a click located on the right side of the wheel indicates a positive valence, and, if the click is higher on the y-axis it suggests higher arousal.

The GEW (Figure 3), developed by Scherer (2005), was used to measure emotions in response to art experiences and it was available (Laurans, 2011). In the past, it has been attested to be useful in identifying the type and strength of the emotions experienced from encounters with artworks in museum studies (Tinio and Gartus, 2018; Schino et al., 2022). In GEW, emotions are systematically aligned in a circle, consisting of twenty emotion families of interest, amusement, pride, joy, pleasure, feeling love, feeling awe, relief, surprise, nostalgia, compassion, sadness, fear, shame, guilt, disappointment, envy, disgust, contempt, and anger. Each emotion was measured in three aspects: *Valence* (positive and negative), *Arousal* (i.e., Control or Power; high and low), and *Intensity* (high and low). In the center of the wheel, the response options of “No” and “Other” were also offered as alternatives. If “Other” was clicked in, participants could type in their answers.

#### Sentiment and semiotic strategies: dyadic interaction

The prompts used during the conversation were based on semiotic strategies to facilitate reflection (Supplementary Table 1A: Appendix A). This kind of interaction with a known peer was incorporated to encourage a naturalistic conversation of art and maintain a feeling of safety and intimacy during the experiment. In line with Van Heusden (2009), we coded from speech contents of the participants’ conversations the following strategies: *perception, imagination, conceptualization* and *analysis*. For sentiment, zero-shot classification sentiment analysis is used to compute emotion scores of the text transcripts of conversational data. This method calculates the probability that a given text aligns with specific emotional classes, providing emotion scores for the text transcripts, in the present case, of conversational data. Through this method we can treat emotions as complex dynamic systems and generate synthetic emotional data (i.e., emotion scores). Specifically, “EGA leverages methods from dynamical systems analysis, specifically the generalized local linear approximation (GLLA) (Boker et al., 2010), to estimate the first-order derivatives of the multivariate data” (Tomašević and Major, 2024, p. 6). Ten participants (five dyads) were removed from the analysis of conversational data because they conducted the dyadic interaction in Dutch, and this study used techniques (such as the zero-shot classification) that were previously validated in the English dataset only. For this reason, we considered only data from English-speaking participants, subsequently using data from 28 participants for the coding of the transcripts and for performing sentiment analysis.

### Data analysis

This rich dataset allows for several pathways of analysis. For the scope of the present study, we proposed the following.

#### Self-report data (quantitative analysis)

Participants provided self-report measures of emotional assessment throughout the study, collected with the GEW. In total, each participant provided eight self-report measures of emotional assessment throughout the study: a maximum of four emotions could be selected in the pre-questionnaires (two regarding their own object and two regarding the other participant’s object) and again a maximum of four in the post-questionnaires. The *x* and *y* coordinates of the points placed on the GEW were used to identify the *Valence*, *Arousal* and *Intensity* of each emotion selected by participants (Coyne et al., 2020). Based on the coordinates of the point we could understand under which quadrant (see Figure 3) the point was located and therefore, if a more positive or negative emotion was reported (*Valence*, x-axis) and if it was characterized by higher or lower levels of *Arousal* (y-axis). The distance from the center was calculated to compute *Intensity.*^5^


$$D\,i\,s\,t\,a\,n\,c\,e=\frac{\sqrt{{\left(x-x_{c\,e\,n\,t\,r\,e}\right)}^{2}+{\left(y_{c\,e\,n\,t\,r\,e}-y\right)}^{2}}-r_{m\,i\,n}}{r_{m\,a\,x}-r_{m\,i\,n}}.$$

Furthermore, we analyzed changes in variability happening along the x-axis (for *Valence*) or the y-axis (for *Arousal*), as these represent distinct constructs. By treating them separately, we preserved their unique contributions instead of reducing the data to a two-dimensional (2D) spatial dispersion, which would obscure potential differences between *Valence* and *Arousal*. Since the data did not meet the assumption of normality, we used the Fligner-Killeen test: a non-parametric and robust test for homogeneity of variances based on ranks (Fligner and Killeen, 1976). This approach allowed us to determine whether changes in variability occurred along a specific axis, aligning with our theoretical expectation that *Valence* and *Arousal* might exhibit distinct patterns of variability. In a separate analysis, we also used the coordinates of the points participants clicked on pre- and post- dyadic interaction during this affective assessment task to compute Euclidean distances and tested the difference through unequal variances Welch’s *t*-test.

#### Conversational data (quantitative and qualitative analysis)

The audio recordings of the dyadic interactions (i.e., conversational data) were first manually transcribed and then coded to identify the four strategies. For this purpose, the coding scheme by van Klaveren et al. (2023) was used as a starting point. This scheme evolved during the iterative coding process as participants introduced new words aligning with the strategies (see Supplementary Appendix C). Additionally, the Cognitive Discourse Analysis (CODA) by Tenbrink (2015) provided inspiration and guidance for developing the general coding framework. We utilized ATLAS.ti Web^6^ as an annotation and coding tool, enabling live collaboration among research group members and, in turn, facilitating researcher triangulation. The research team achieved intersubjective agreement by collaboratively analyzing data or systematically reviewing and discussing the coding with colleagues (Levitt et al., 2017). Specifically, one researcher (i.e., the first author) conducted the primary coding work, while two other researchers reviewed each transcript and coding in-meeting-sessions. Additionally, the rest of the research team randomly checked singular transcripts of random dyads as a final reliability-check. This process ensured rigor in coding from the development of the codebook (process) to the creation of the code manual (product, Supplementary Appendix C; Denzin and Lincoln, 2018). The qualitative data (conversational data) was first coded into sentences that aligned with one of the four semiotic strategies. Each of these coded sentences was then analyzed through EGA, where the latent network structure of sentiments was revealed.

To this end, the coded sentences were analyzed utilizing the *transforEmotion* R package (Christensen et al., 2022; version 0.1.4). This package employs Generative AI, specifically Transformer Models, to perform sentiment analysis via Hugging Face’s^7^ zero-shot classification model pipelines for text, images, and videos. In this analysis, the pipeline “MoritzLaurer/deberta-v3-base-zeroshot-v2.0” was selected due to its training on zero-shot classification specifically for English text, where it classifies text according to possible use-case categories provided by the researcher (Laurer et al., 2023). We then used Exploratory Graph Analysis (EGA) (Golino and Epskamp, 2017) through EGAnet R package (Golino and Christensen, 2019) to model sentiment dynamics and reveal the complex interrelationships between Sentiment and Strategies. EGA reports the number of nodes (representing variables, in our case sentiments), edges (unique associations, that is, partial correlations between nodes), edge density, and descriptive statistics about these edges, which help describe the network’s structure. EGA uses network analysis techniques to detect clusters, or latent communities (i.e., dimensions), based on the statistical co-occurrence patterns of the nodes. To investigate the relationship between strategies and affect, statistical models containing both fixed effects and random effects have been proposed. More specifically, a Linear Mixed-Effects Model (LMM) comparing aspects of perceived affect before and after the dyadic interaction was fitted with the lme4 (Bates et al., 2003) R package. Lastly, a Multinomial Log-linear Model (MLM) investigating the sentiments in combination with the semiotic strategies during the dyadic interaction as predictors for perceived affect was fitted with the nnet (Ripley, 2009) R package. Precise p-values were computed with Kenward-Roger approximation for the degrees of freedom through pbkrtest and sjPlot R packages (Halekoh and Højsgaard, 2011; Lüdecke, 2013; Nakagawa et al., 2017).

## Results

### Changes in perceived affect after the dyadic interactions

To test whether the dyadic interaction influences the *Perceived affect* toward the art objects in terms of *Valence*, *Arousal* or *Intensity* (H1), we compared the differences between the points placed on the Geneva Emotion Wheel (GEW) (Scherer, 2005) by the participants to indicate their affective responses regarding their own [*own*] objects and the objects belonging to their co-participant [*other*].

We begin with a broad overview of the phenomenon at the group level, followed by a more in-depth exploration through exploratory analyses that account for individual differences, allowing for a nuanced understanding of differences across participants. For the group level analyses, the totality of 304 points, placed on the GEW was used, regardless of whether they were placed inside or outside of the wheel as we focus on the information conveyed by the *Valence* (*x*) and *Arousal* (*y*) axes (see Methods for further details). In the individual-level analysis, we focused on the *Intensity* of emotions reported using the GEW. To ensure that the intensity was measured meaningfully, we included only the points that were selected within the wheel and thus explicitly specified intensity, this means points on the label of the emotion and the ones within “None” or “Other” sections (respectively, 0 points for “None” and 4 points of “Other” in the entire study) were excluded by this analysis for a total of 196 points.

#### Valence and arousal (group level)

**FIGURE 4:**
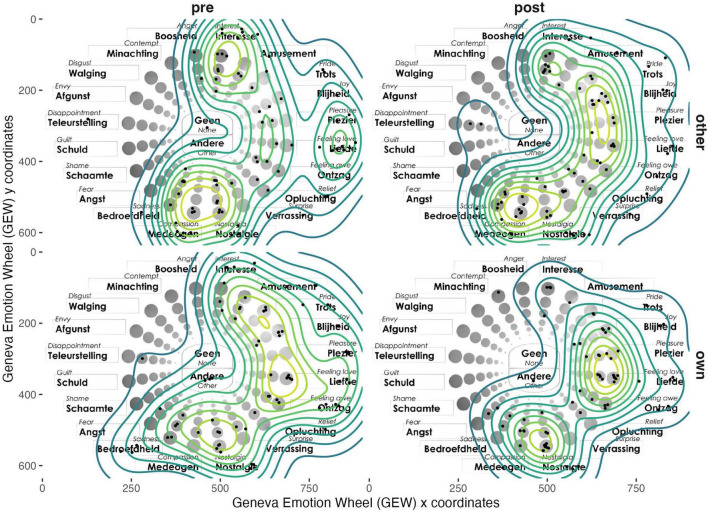
Contour plot of distribution of points in Geneva Emotion Wheel (GEW).

The changes in similarity between the members of the dyads, in terms of *Valence* (x-axis) and *Arousal* (y-axis), before and after the conversation were analyzed with Fligner-Killeen tests. For *Valence*, the test approached statistical significance (med χ^2^ = 3.521, *p* = 0.060), indicating reduced variability between dyad members after the conversation. This suggests that participants’ *Valence* are not equal and, as shown in Figure 4, they became more aligned, reflecting a narrowing in the range of emotions related to (un)pleasantness. In contrast, for *Arousal*, the test did not reveal a significant change in variability between dyad members (med χ^2^ = 1.800, *p* = 0.179). This indicates that *Arousal* (ranging from calm to excited) did not show greater alignment before and after the conversation.

These findings suggest that while participants’ emotional valence aligned almost significantly toward the same type of emotions at the group level, the levels of arousal of these emotions were not significantly affected. Additionally, we can see in Figure 4 that the points on the GEW are more clustered around the positive emotions of *Joy*, *Pleasure*, *Feeling Love* and *Nostalgia* after the dyadic interaction. On the other hand, the points reported before the conversation are more scattered around the emotion wheel, displaying additional hotspots around different emotions, like *Compassion* or *Interest*, as well as disseminated points around *Sadness* or *Disappointment*.

#### Art objects (group level)

We calculated how much the distances between points placed on the GEW per object within each participant changed from the first measurement (pre-dyadic interaction) to the second measurement (post-dyadic interaction). A Welch’s *t*-test was used to compare these distances due to its robustness against unequal variances and unequal sample sizes between the two groups. The analysis revealed a statistically significant difference in distances between their own and the other’s object, *t*(167.01) = -1.98, *p* = 0.049 The mean difference for the “own” object (*M* = 214) was lower than for the “other” object (*M* = 249). This suggests that participants’ GEW responses concerning their own object remained more consistent before and after the conversation, while their perceptions of the other person’s object showed greater change at a group level.

#### Exploratory analysis: impact of valence, arousal, object on intensity (individual level)

In order to gain deeper insights, we conduct an exploratory analysis that examines the influence of *Art objects*, *Valence* and *Arousal* on *Intensity* of the emotions reported in the GEW at the individual level. We therefore included both participants [*pid*] and dyads [*dyad_id*] as random factors. By considering these variables, we aimed at capturing the nuanced variations of how individual differences influence *Perceived affect* toward art.

The *Intensity* score was calculated by measuring the distance from the center of the wheel, and then normalized to range between 0 and 1, treating intensity as a continuous variable (see Materials and methods section for further details) We applied Linear Mixed-effects Models (LMM) to predict the *Intensity* of the emotions reported on the GEW with various predictors, including the *pre_post* (the time variable, where [*pre*] is before the conversation and [*post*] is after the conversation), *object* (this takes into account the art object of reference being either [*own*] or [*other*]), *Valence* of emotion ([*positive*] or [*negative*]), *Arousal* of emotion ([*low*] or [*high*], and *Quadrant* (combinations of valence and arousal into: negative valence/low arousal [*neg_lowAr*], negative valence / high arousal [*neg_highAr*], positive valence/low arousal [*pos_lowAr*], positive valence / high arousal [*pos_highAr*]). We accounted for individual differences by including random effects for both participant identifiers [*pid*] and dyad identifiers [*Dyad_id*], capturing the nested structure of the data (see Table 1).

**TABLE 1:** Overview of mixed-effects models on intensity of reported emotions.

	Basic model				Arousal model				Valence model				Valence and arousal model				Quadrant model			
**Predictors**	**Esti** **mates**	**CI**	***p***	**df**	**Esti** **mates**	**CI**	***p***	**df**	**Esti** **mates**	**CI**	***p***	**df**	**Esti** **mates**	**CI**	***p***	**df**	**Esti** **mates**	**CI**	***p***	**df**
(Intercept)	0.75	0.68–0.82	**< 0.001**	37.48	0.76	0.69–0.83	**<0.001**	45.00	0.73	0.63–0.82	**<0.001**	80.22	0.73	0.64–0.82	**<0.001**	82.90	0.70	0.52–0.87	**<0.001**	186.11
Pre post [post]	0.03	–0.03 to 0.08	0.318	179.71	0.03	–0.03 to 0.08	0.327	178.73	0.03	–0.03 to 0.08	0.324	178.40	0.03	–0.03 to 0.08	0.336	177.29	0.03	–0.03 to 0.08	0.337	176.29
Object [other]	–0.09	–0.15 to –0.03	**0.005**	1920.23	–0.08	–0.14 to –0.02	**0.006**	190.90	–0.08	–0.14 to –0.02	**0.006**	190.50	–0.08	–0.14 to –0.02	**0.010**	188.48	–0.08	–0.14 to –0.02	**0.010**	187.40
Arousal [high]					–0.03	–0.08 to 0.03	0.360	184.47					–0.03	–0.09 to 0.02	0.244	182.72				
Valence [positive]									0.03	–0.04 to 0.10	0.407	188.48	0.04	–0.03 to 0.12	0.275	186.82				
Quadrant [neg_lowAr]																	0.03	–0.14 to 0.20	0.712	180.11
Quadrant [pos_highAr]																	0.04	–0.13 to 0.20	0.642	180.09
Quadrant [pos_lowAr]																	0.07	–0.09 to 0.24	0.375	180.06
N	29 _pid_				29 _pid_				29 _pid_				29 _pid_				29 _pid_			
	17 _Dyad_id_				17 _Dyad_id_				17 _Dyad_id_				17 _Dyad_id_				17 _Dyad_id_			
Observations	196				196				196				196				196			
M./C. R^2^	0.045/0.255				0.048/0.251				0.047/0.263				0.052/0.260				0.051/0.259			

neg_lowAr, negative valence/low arousal; neg_highAr, negative valence/high arousal; pos_lowAr, positive valence/low arousal, pos_highAr, positive valence/high arousal. M. R^2^, Marginal R^2^, variance of the fixed effects. C. R^2^, Conditional R^2^, variance of both the fixed and random effects. Reference levels per variable: *pre_post*: pre; *object*: own; *Valence*: negative, *Arousal*: low; *Quadrant*: neg_highAr. Bold type was used to highlight significant *p*-values (less than or equal to the significance level ≤0.05).

When taking into account the individual level as in these models, *pre_post* is consistently non-significant across models. We can also notice that *object (own)* is consistently positive and significant across all models, suggesting that participants always show greater *Intensity* for their own objects compared to what the other person brought. Neither *Valence* nor *Arousal* had significant individual effects on emotional *Intensity*. Moreover, their interaction as represented by the *Quadrant* variable (combinations of *Valence* and *Arousal*) did not significantly impact the *Intensity* of emotions reported. This suggests that, within the context of this study, the intensity of the emotion was not strongly driven by whether the emotion was negative or positive, nor by its associated arousal level.

### Relationship between affective aspects and semiotic strategies during dyadic interactions

To test whether sentiment (as Affective aspects of the experience) differs between semiotic strategies, we made use of both quantitative and qualitative data drawn from the dyadic interactions (i.e., conversations). The zero-shot sentiment analysis was based on seven emotional classes that are the most recurrent self-reported (GEW) categories of emotions: *Sadness, Enjoyment, Compassion, Awe, Interest, Joy, Nostalgi*a and *Love*. These categories were the most chosen in the entire study as they account for the 69.97% of all the points reported on the wheel with frequencies, respectively of: 20, 22, 24, 25, 27, 30, 39, 45; while the remaining categories had 15 counts or less.

In order to have a more in-depth look at what happens within each of the Strategies, we performed an Exploratory Graph Analysis (EGA) (Golino and Epskamp, 2017) and retrieved latent communities using network scores.

**FIGURE 5:**
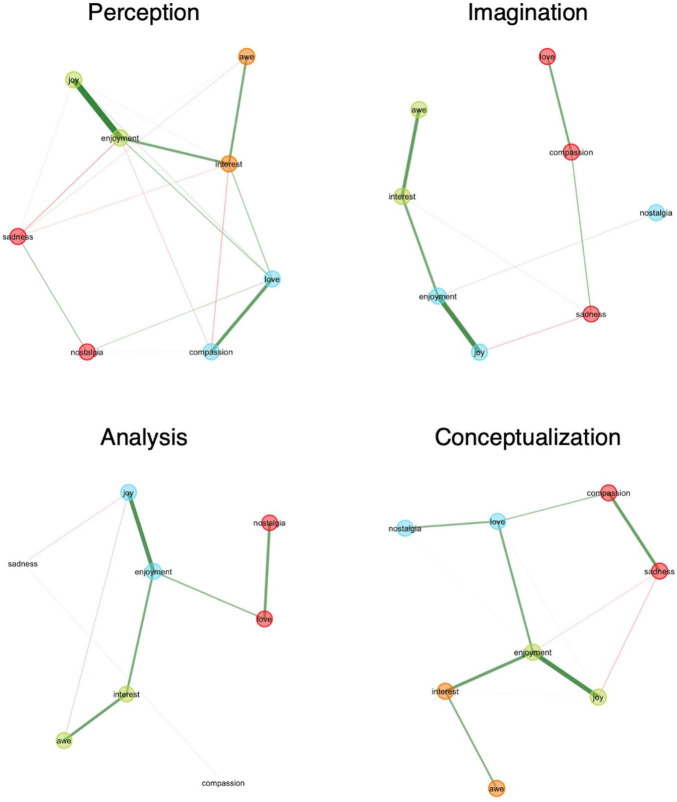
EGA plot of latent communities of sentiment across strategies. Nodes are positioned based on the eigenstructure of the adjacency matrix (Golino and Christensen, 2019), which organizes them according to the strength and pattern of their connections. This layout brings closely related nodes nearer to each other.

The graphs in Figure 5 illustrate connections between variables (nodes) represented as lines (edges), with line thickness indicating the strength of the correlations. Nodes are color-coded, based on the communities assigned by the bootstrapped EGA algorithm. The EGA (overview in Supplementary Table 2D: Appendix D) revealed three distinct latent communities of Sentiment for *Imagination* and *Analysis* and four in the case of *Perception* and *Conceptualization*. When analyzing the EGA results (see Figure 5), the negative correlations (red lines) reveal a common pattern which is evident across all strategies, in particular a persistent negative correlation between *Sadness* and *Joy*. The positive correlations (green lines) between *Joy* and *Enjoyment and Interest* and *Awe* are consistently observed across all strategies. The case of *Nostalgia* is rather unique, as it exhibits a strong correlation with *Love* during *Conceptualization* and *Analysis* but is almost entirely disconnected from other emotional classes in the case of *Imagination*, with only one positive correlation with *Enjoyment*.

Notably, the nodes (classes of sentiments) identified in sentiment analysis appear to be more interconnected during the *Perception* strategy than in any other, supporting our earlier findings. The differences in *sentiments* observed specifically within the *Perception* strategy, combined with this interconnectedness, suggest that this cognitive approach elicits a unique sentiment profile.

When testing the stability of the EGAs, we found that overall, the dimensions are relatively stable across the *strategies*, with the exception of *Sadness* and *Compassion* in *Analysis*. This means that configural invariance –the consistency of dimensions across groups– is not fully established. For this reason, we removed items with replication less than 0.50 in their assigned community as they are considered unstable and therefore not invariant. Accordingly, only partial configural invariance is met.

To take this analysis a step further, *sentiments* were also analyzed for metric invariance –which examines whether specific sentiments differ significantly across strategies by comparing their mean levels– as per protocol by Jamison et al. (2024). In this analysis, multiple pairwise comparisons were conducted, and the *p*-values were adjusted using the Benjamini-Hochberg (BH) method to control the false discovery rate (FDR), ensuring that the results are not inflated due to the number of comparisons. To this end, we will hereby interpret only significant *p_BH* values (*p*-values that went through FDR correction), while in Supplementary Table 1D: Appendix D both raw *p*-values (*p*) and adjusted *p*-values (*p_BH*) are provided.

*Joy* and *Enjoyment* exhibited stronger connections in the *Perception* strategy compared to the *Conceptualization* strategy (both *p_BH* = 0.032). When considering raw *p*-values, additional significant differences emerged in comparisons involving *Perception*, suggesting that this strategy is notably distinct in terms of its associated sentiments compared to others. For instance, significant differences were found for nostalgia (*p_BH* = 0.016) and compassion (*p_BH* = 0.041) when comparing *Imagination* and *Analysis*. *Nostalgia* showed a stronger connection in the *Analysis* strategy, whereas compassion was more strongly associated with the *Imagination* strategy. These results reveal significant differences in *Sentiment* across the *Strategies*, confirming that some emotions are more predominant than others in specific strategies. Our analysis aligns with H2, which states that the affective aspects and semiotic strategies are intertwined with one another during the process and that significant differences in sentiment emerge across the four *Strategies*.

### The influence of affective aspects and semiotic strategies on the perceived affect after dyadic interaction

To test the influence of *Sentiment*, *Strategies* and their interaction on *Perceived affect* (3), we ran a Multinomial Log-linear Model to analyze a categorical dependent variable with four levels, namely the four *quadrants* of the GEW (Supplementary Table 3D: Appendix D). To avoid distortion of the results, we decided to simplify and trim the model by removing sadness, compassion, and enjoyment. This operation was undertaken because (1), these emotions were, among the 8 selected ones, the least reported ones, potentially suggesting that they may not be as relevant; (2) the EGA invariance analysis revealed that sadness and compassion in Analysis were the most unstable construct and could confound the results; (3) upon testing multicollinearity, we found high Variance Inflation Factors (VIFs) for sadness (*VIF* = 23.646) and enjoyment (*VIF* = 21.524), suggesting that these variables are highly correlated with others in the model, perhaps because of the overlap with other sentiments—for example, in the case of enjoyment, interest, love or joy (James et al., 2021; Bruce and Bruce, 2017).

This model allowed us to evaluate the effects of predictors (*Sentiment*, *Strategies*, and their interactions) on the likelihood of falling into each *Quadrant* (relative to the reference level). The reference level for the *Quadrant* variable for comparison is high arousal—negative valence [*neg_lowAr*], due to its less frequent selection, making it a good baseline for comparisons. The *Perception* strategy serves as the reference level against which the other semiotic strategies [*Imagination*, *Conceptualization*, and *Analysis*] are compared as it is the most concrete and basic of the four strategies. This means that any coefficients for these strategies represent the difference in self-reported emotional outcomes relative to the perception strategy.

#### Sentiment and strategies influences quadrant after dyadic interaction (main effect)

Positive classes of *Sentiment* (i.e., *Joy* and *Interest*) have significant effects on the likelihood of falling into different *quadrants* of *Perceived affect*. The probability of *Joy* during the dyadic interaction is significant in increasing the chance of both high-arousal (β = 0.47, *p* = 0.022, 95% CI [0.07, 0.88]) and low-arousal (β = 0.44, *p* = 0.34, 95% CI [0.03, 0.84]) positive states.

#### Strategies influence quadrant after dyadic interaction (main effect)

None of the *Strategies* has a significant effect on the likelihood of falling into different *quadrants* of *Perceived affect*. Thus, it is important to notice that *Strategies* alone cannot predict the emotional outcome self-reported by the participants.

#### Sentiment*strategies influence quadrant after dyadic interaction (interaction effect)

*Sentiment* and *Strategies* show significant interaction effects. For instance, the interaction between *Conceptualization* and *Joy* implies that when people use conceptual strategies and experience a joyful sentiment during reflection seems to be a constant significant predictor for all the levels of the response variable *Quadrant* (neg_lowAr: β = –0.57, *p* = 0.049, 95% CI [–1.14, –0.00]; pos_highAr: β = –0.83, *p* = 0.003, 95% CI [–1.38, –0.28]; pos_lowAr: β = –0.76, *p* = 0.007, 95% CI [–1.30, –0.21]). Similarly, the same can be said for the combination of *Conceptualization* and *Interest*: this combination can predict when people are going to report a negative low-arousal (β = 0.66, *p* = 0.070, 95% CI [–0.05, 1.370] approaching significance) and high-arousal positive (β = 0.83, *p* = 0.019, 95% CI [0.14, 1.53]) states alike. Similarly, the interaction between *Imagination* and *Interest* approaches significance (β = 0.48, *p* = 0.079, 95% CI [-0.06, 1.02]) in predicting people’s positive high-arousal affect. *Analysis* and *Joy* was significant across multiple levels of *Quadrant*, including neg_lowAr (β = –1.23, *p* = 0.041, 95% CI [–2.40, –0.05]), pos_highAr (β = 0.22, *p* = 0.033, 95% CI [0.05–0.89]), and pos_lowAr (β = –1.24, *p* = .038, 95% CI [–2.41, –0.07]).

Therefore, the present analysis rejects H3b, proving that *Strategies* alone does not predict the *Perceived affect* by the participants after the dyadic interaction. However, we found minimal support for H3a and partial support for H3c, since only specific classes of sentiment and their interaction with semiotic strategies significantly predicts the *Perceived affect* of participants after the dyadic interaction.

## Discussion

### Changes in perceived affect after the dyadic interactions

Our findings show that participants’ emotional valence aligned almost significantly post interaction, indicating reduced (un)pleasantness of the emotion. On the other hand, emotional arousal remained equally distant between dyad members. This confirms previous literature that points out the independence of arousal and valence, as evidenced by the enjoyment of horror films (Hanich et al., 2014; Menninghaus et al., 2017). This insight adds up with the mixed result of empirical studies trying to test arousal theories (for an overview, see Zsidó, 2024). In line with Storbeck and Clore’s “affect as information” theory (Storbeck and Clore, 2008), arousal does not necessarily fluctuate based on external events, but rather influences the processes behind understanding by signaling the salience of emotional responses. This confirms previous findings about the role of arousal, since “modern research on interest assigns no role to arousal” (Silvia, 2005b, p. 353). Valence, on the other hand, was more susceptible to change as participants re-evaluated or reframed their affective responses in relation to the art object (i.e., artwork) during the interaction. More specifically, Knoll et al. (2024) showed that repetition of art experiences may play an important moderating role especially on valence as affective measure, while differences in arousal level were detected only by repetition of highly beautiful stimuli (art objects as well as experiencing surrounding and nature). That is, the novelty processing (Sander et al., 2018), likely played a role in the emotional shifts observed after the dyadic interaction. In this study, participants may have experienced the other person’s object as novel or unfamiliar, which could have led to re-evaluations of its significance as it became an integral aspect of the art experience itself.

On the one hand, this suggests that the dyadic interaction is influencing the experience of unfamiliar art objects. On the other hand, when accounting for participants as a random factor, this effect diminished, indicating variability in how individuals emotionally engage with others’ art objects. Inter-individual differences play an influential role in people’s experiences and Jacobsen’s framework for the Psychology of Aesthetics (Jacobsen, 2004, 2006) offers a useful lens to understand this variability in the specific context of art experiences. This supports the idea that affective responses to art can greatly differ between participants and are shaped by (individual and collective) memory and prior experiences.

Additionally, personal relevance –whether the art object belonged to the participant or someone else– seems to amplify emotional intensity, as previously found in literature too (Brosch and Sharma, 2005; Sutherland and Mather, 2018). Building on this, incorporation of participants’ personal selection of art objects, combined with tailored instructions, allowed us to capture a more authentic response, especially in a lab-based environment. However, this approach also introduced a potential limitation, as it reduced our control over the nature of the art objects. For instance, the characteristics of the art objects (such as the form, that is, whether a visual artwork, musical piece, or other) may have contributed to less consistent findings. For example, the specific form of the art object, as well as the level of personal relevance, could play a role in how the emotional responses are structured. For example, Knoll et al. found that “people appear to feel calmer during more beautiful experiences” (Knoll et al., 2024, p. 7), hinting that arousal levels may differ depending on the beauty of what people are experiencing. However, we prioritized the process over the stimulus, deliberately choosing to focus on the art experience itself and not on artworks as physical objects with universal properties (e.g., beauty) that distinguish them from other kinds of objects (Hayn-Leichsenring, 2017). We aligned with the notion that art is best understood through the experience it provokes rather than the object it comes with. As Specker (2023) notes, the stimulus-oriented approach may overlook the processes that constitute our experience with art. This has also been outlined by Nadal and Skov (2024), who refer to experiences with art as “not solely determined by stimulus properties, but are substantially shaped by the agent’s learned experience, physiological state and ongoing behavioral circumstances.” By adopting a process-based perspective –such as Dewey’s understanding of art as experience (Dewey, 1934) or Van Heusden’s theory of art as reflective imagination (Van Heusden, 2009)– we can move beyond institutional definitions of “art” and focus on the functions and impacts of art in our lives, looking at it as a tool for reflection and destabilizing the traditional distinctions between “high” and “low” art.

### Relationship between affective aspects and semiotic strategies during dyadic interactions

**FIGURE 6:**
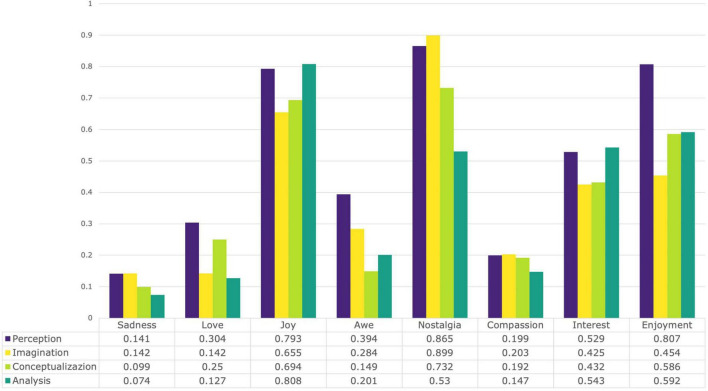
Means for probabilities (0-1) of sentiment across strategies.

The findings indicate that each semiotic strategy uniquely engages with sentiments during the dyadic interactions involving art objects. The graph in Figure 6 reveals interesting trends in the probabilities of emotions across different strategies. Overall, concrete strategies exhibit higher probabilities of emotions compared to abstract strategies. Nostalgia is most prominent in more concrete semiotic strategies (perception and imagination), followed by enjoyment in perception and joy in imagination. Additionally, the likelihood of sadness and nostalgia diminishes as strategies become more abstract. There are many significant differences in terms of means of probability of sentiment occurring in each strategy and the Exploratory Graph Analysis (EGA) revealed different edge densities and intercorrelations of sentiment within strategies.

Perception particularly stands out for significant differences observed in sentiments like love, awe, nostalgia, and interest compared to the other strategies (0.394, see Figure 6), suggesting that this sensoric and concrete strategy evokes a distinct and intricate sentiment profile when mediated by an (art) object. For example, there is a higher chance, on average, that the sentiment of awe occurs within this strategy compared to any other, corroborating the results from the language analysis by Darbor and colleagues reporting that awe is “related to observing the world, reflected in greater use of perception words” (Darbor et al., 2016, p. 1188). Within the network grasped by the EGA, awe is clustered together with interest, and it is negatively correlated to sadness. In all the other strategies’ networks, awe is actually only positively correlated with interest, or joy (only in the case of analysis). It seems only in the case of perception there are more complex connections, perhaps because perception is the strategy that shows the most dense and intricate network (with an edge density of .643). Its high edge density suggests that emotions are more integrated and interconnected during Perception than in any other strategy. This may suggest that when individuals focus on the direct, sensory experience of an (art) object, there is a strong integration of different sentiments. In contrast, imagination and the other strategies involve more semiotic processing, adding new layers of interpretation and symbolic meaning. This suggests that sensory engagement with an art object –whether visual, auditory, or tactile– can enrich the integration of affect into the art experience. This integration is not solely dependent on the individual but is also shaped by the form and qualities of the (art) object itself, highlighting the dynamic relationship between the viewer and the artwork.

We find the moderate edge density (0.429) of conceptualization: this network’s edge density suggests moderate emotional integration within conceptualization, where emotions are not as closely tied as in perception but are more interconnected than in imagination or analysis. Conceptualization allows for the categorization, interpretation, and valuation of the art experience. While it follows upon the initial sensory engagement, it also has the potential to inform or enrich the experience, as when additional information –such as the intentions or context– is brought into the reflection. Nostalgia is the sentiment that is most likely to occur within conceptualization, although it was indeed more prominent in the concrete strategies, imagination and/or perception. This implies that nostalgia may be triggered more by concrete experiences like musical ones (Barrett et al., 2010), tastes (Reid et al., 2023), or temperature (Zhou et al., 2012) rather than by more abstract processing (Juhl et al., 2010). The EGA reported that, while nostalgia is always found to be interconnected with love during conceptualization and analysis, it emerged as an almost disconnected node in the network of imagination (only feebly connected to enjoyment). We could hypothesize nostalgia, as it is commonly defined as “characterized by fond reflection on past events” (Juhl and Biskas, 2023, p. 1), emerges only during the use of abstract semiotic strategies, whereas imagination triggers nostalgia’s peripheral features like daydreaming, and desire (Hepper et al., 2012). Central features of nostalgia tie more directly to concrete experiences from the past rather than to new, hypothetical constructions that are typical of imagination. Therefore, interrelation of nostalgia with other sentiments shaping the strategies is less evident in imagination.

The sentiment analysis detected three more distinct communities in the EGA network of imagination –one consisting of sadness, love, and compassion; another of joy, enjoyment and nostalgia; and the last of awe, and interest. The low edge density detected in the network of imagination (0.286), implies that classes of emotions are less interconnected. Since imagination is the semiotic strategy upon which the art experience relies the most, this finding may indicate that art experiences generate a less structured emotional landscape. This could be because imagination reshapes emotional patterns, much like how imaginative thinking creates new realities from the sensory input provided by perception (van Dorsten, 2015; Stamkou et al., 2024).

Similarly to imagination, analysis showed a lower edge density (0.286). It shows three clusters: one encompassing joy and enjoyment, another comprising awe and interest, and a third one composed of love and nostalgia. Additionally, it showed isolated nodes of sadness and compassion, which do not fall under any cluster. This may suggest that sadness and compassion may play distinctive roles in analytical processes. This aligns with findings that sadness is often elevated in analytical, self-focused thinking in contexts besides art experiences. Research indicates that sadness promotes analytical and systematic processing of information (Forgas, 2003). However, our results show that, in analytical strategies during art experiences, sentiments like sadness and compassion appear to be disconnected from other emotions, except for a faint mutual connection and sadness, a weak negative correlation between sadness and joy.

The consistent clustering of joy and enjoyment and awe and interest across all of the strategies could indicate that these emotions are central to experiences of art, while a stronger connection of sadness and nostalgia with the rest of the network can be observed as typical of abstract strategies (Christensen et al., 2023b). While these interpretations may offer a meaningful lens through which to understand the nature of emotions elicited through art, they remain speculative and should serve as a starting point for further investigation.

Worth noting is that all the semiotic strategies are multidimensional (more dimensions or clusters have been detected), highlighting the complex interplay between emotions. This finding supports the 5E approach, which stresses the importance of the whole body’s intra- and inter-corporeal interactions and phenomenological experiences in shaping a deeper understanding of the world (Troncoso et al., 2023). According to this approach, understanding is embodied, embedded, enacted, extended, and emotional (Varela et al., 1991; Thompson, 2007; Colombetti, 2014; Newen et al., 2018; Pérez and Gomila, 2022). This has two key implications: first, the fact that emotions are integral to all four semiotic strategies suggests that affect is a fundamental component of the art experience, rather than a mere byproduct of it; second, participants are not passively experiencing emotions but are actively constructing them and their experience through interactions with their social and physical environment (Caruana and Gallese, 2012). The current study primarily focused on the exploration of affective aspects, whereas future research could investigate how other dimensions of the 5E approach –particularly embodiment and enaction– contribute to the experience (Drummond, 2024).

### The influence of affective aspects and semiotic strategies on the perceived affect after dyadic interaction

Lastly, the interaction between affective aspects and semiotic strategies has been tested with a Multinomial Log-linear Model. The interaction revealed significant influences on the perceived affective state after the dyadic interaction. This result brings us an important insight: semiotic strategies alone are not predictive of a final emotional outcome but only in combination with affective aspects of the art experience.

For example, our findings revealed the interaction effects in the Multinomial Model between conceptualization and analysis with joy and interest as stable and reliable predictors of perceived affective states. The interaction between conceptualization and interest shows how abstract thinking processes, such as categorizing (thus, relying on conceptual strategy), may amplify positive, high-arousal emotional states. This is not surprising, given that interest –a feeling associated with curiosity, exploration, and intrinsic motivation– holds a central place in the psychology of art (Tan, 2000; Silvia, 2005b; Pelowski et al., 2017). It stimulates exploration and promotes engagement with artworks (Silvia, 2005a). Gernot et al. (2018) suggest that heightened interest can explain positive feelings (both high- and low-arousal) toward negative artworks (such as sad music), due to its association with emotional contagion, which may have been triggered by the dyadic interaction and overall design of our experiment. However, it is important to notice that it is only when it interacts with conceptualization that it becomes a significant predictor of perceived affect. Magon and Cupchik suggest that interest can often lead to deeper exploration of self-identity (Magon and Cupchik, 2023). We might then infer that the interaction between conceptualization and interest leads to a clearer understanding of the perceived affective outcome, particularly in the case of high-arousal positive states.

This perspective aligns with the definition of transformative experiences as given by Pizzolante et al. (2024), where transformative experiences are indeed characterized by enhanced interest. These experiences are seen by some scholars as resulting from a misalignment between pre-existing cognitive schemas and encounters with novelty, resulting in cognitive “disequilibrium,” which might trigger emotional responses. As Piaget (1977, p. 275) explains, “On the one hand, the reciprocal assimilation of schemata and the multiple accommodations which stem from them favor their differentiation and consequently their accommodation; on the other hand, the accommodation to novelties is extended sooner or later into assimilation, because, interest in the new being simultaneously the function of resemblances and of differences in relation to the familiar, it is a matter of conserving new acquisitions and of reconciling them with the old ones”. According to van Dorsten (2015), perception and analysis unfold through the accommodation to the environment, while imagination and conceptualization assimilate it (imagination creates something new, but conceptualization “tames” the new by labeling it within terms of existing concepts). This back-and-forth between assimilation and accommodation helps explain why the interaction of interest with conceptualization and joy with both conceptualization and analysis are significant predictors of perceived affect. Joy has indeed a “broadening and building” effect, encouraging engagement with the environment in both an assimilating and accommodating way (Johnson, 2020).

As Pizzolante et al. (2024, p. 4) note, “These affective responses can range from initial confusion or frustration to feelings of e.g., satisfaction, joy, or even awe, upon resolving the dissonance”. This supports the idea underlying the present research that affects (in this specific case, joy and interest) are not merely byproducts of art experiences but active components in them. Their interplay with semiotic strategies leads to insights and epiphanies –possibly influencing the final affective outcome, as suggested by our findings. Frijda (1988, p. 350) “Law of Situational Meaning” further supports this idea: “In the emergence of emotions people need not be explicitly aware of these meaning structures. They do their work, whether one knows it or not.”

The interplay between strategies and sentiment during dyadic interactions with art reveals a complex yet insightful relationship between affective aspects and semiotic strategies, particularly the role of conceptualization in shaping emotional outcomes (Perceived affect) during art experiences. This could be due to the fact that, when language and dialogue are employed, conceptualization constitutes the dominant underlying strategy. Although promising, these results were inconclusive and warrant more attention in future studies, potentially pointing at the inclusion of an embodied and enactive direction.

### Strengths and limitations

In our study, we make use of personally meaningful art objects, which, while surely providing unique insights, may simultaneously challenge and enhance the generalizability of our findings. On one hand, the strong personal connections with these objects likely intensified the intertwining of affect and cognition, possibly leading to responses not fully representative of everyday art experiences. The range of media of the art object discussed during these interactions likely shaped the co-occurrence of emotions and strategies, as different forms of art (e.g., visual vs. auditory) may evoke unique emotional and cognitive dynamics and future direction could focus on the multimodal experiences (Clemente et al., 2024). On the other hand, this approach also served as a strength, enabling us to capture a broad spectrum of emotional and cognitive responses that go beyond conventional art (object) appreciation, providing a richer understanding of personally relevant art experiences. Thereby, we do not rely on pre-selected (art) objects that may not provoke art experiences for individual participants. By doing so, we avoided imposing external definitions of what constitutes “art” and focused on the goal of understanding the processes behind art experiences rather than object perception. Theoretically, this approach (more process- than stimulus-oriented) challenges essentialist views that define artworks as physical objects possessing universal properties (e.g., beauty) that differentiate them from other objects (Hayn-Leichsenring, 2017).

Additionally –and remarkably– the present study is one of the few that tried to capture the experiences of emotions like love and compassion in response to art (Stamkou et al., 2024), and more specifically, during conversations about art. The structured, prompt-based nature of the dyadic interactions might be seen as another limitation, potentially constraining the spontaneity of responses and affecting the natural flow of conversation. Moreover, our reliance on verbal data collection might have potentially overshadowed non-verbal forms of engagement (De Jaegher and Di Paolo, 2007; De Jaegher, 2013; Darbor et al., 2016). However, this approach provided valuable insights into how participants articulate and make sense of their experiences, which lays a foundation for future studies to incorporate non-verbal methods, such as drawing or movement, to capture a broader range of embodied responses to art. This structured design, however, ensured that participants engaged with a comprehensive range of semiotic strategies, allowing us to systematically explore how individuals interpret art across varied emotional and cognitive dimensions.

The composition of our sample provides further context to our findings. Participants were primarily adults within a similar age range, with each selecting a co-participant, which could have introduced shared cultural references and heightened emotional dynamics such as nostalgia (Jacobsen and Beudt, 2017). These shared characteristics may have shaped the emotional reactions observed, limiting our understanding of how individual differences across a broader demographic spectrum could influence art engagement. However, it is worth noting that, usually, people normally tend to talk about art with family, friends or colleagues who share the same cultural frames of reference. This naturalistic element reinforces the ecological validity of our approach, even as it highlights the importance of exploring diverse perspectives and contexts in future research, such as accounting for individual differences in mood or personality, which are known to impact emotional processing (Mueller and Kuchinke, 2016; Fraga et al., 2021).

Future research should build on these insights by including participants from varied age groups and cultural backgrounds would help clarify how individual and shared experiences shape emotional and cognitive responses to art. Integrating measures of personality, mood, and interpersonal dynamics within dyads could reveal additional insights into how personal and contextual factors influence the art experience. Exploring multimodal art forms, such as auditory or tactile media, could provide a more nuanced picture of how different sensory channels affect the experience and understanding of artworks (Clemente et al., 2024). Finally, incorporating non-verbal methods like drawing, movement, or other expressive behaviors would offer a fuller picture of how people engage with art beyond verbal reflection. Collectively, these directions would deepen our understanding of the complex interplay between affect, cognition, and culture in art-related experiences, with valuable implications for theory development and educational practice.

## Conclusion

The present research explored the intricate relationships between affect and semiosis, particularly beyond the traditional lab setting, where more personal engagement with art objects occurs. Our multi-method approach revealed the complex, multifaceted nature of participatory art experiences of conversations about meaningful art objects, and the significant influence these interactions have on perceived affect in art experiences.

Results indicate that, at the group level, participants reported notable changes in their perceived affect toward the artwork of the partner after the conversation. Exploratory Graph Analysis revealed that different semiotic strategies engage with emotions in distinct ways, with perception and conceptualization characterized by tightly interconnected emotions, compared to the more diffuse networks in imagination and analysis.

Furthermore, while semiotic strategies help individuals understand sensory input, they alone do not predict emotional outcomes. Notably, when paired with joy or interest, conceptualization and analysis increased the likelihood of positive high- and low-arousal states. We propose that this combination stimulates connections to the artwork, helping individuals engage with it and ultimately bringing further clarity to their perceived affective experiences, especially in the case of positive ones.

Ultimately these findings highlight the mutually supportive interaction between affective aspects and strategies used in shaping meaning with art – and, in turn, transformative experiences.

## Data Availability

The anonymised raw data supporting the conclusions of this article will be made available by the authors, without undue reservation.

## Footnotes

1 G*Power (Faul et al., 2007) for a repeated-measures ANOVA with a medium effect size (*f* = 0.25), α = 0.05, and power (1-β) = 0.80, yielded a required sample size of 34 participants. The final sample of 38 participants exceeded this requirement. As far as qualitative analysis goes, data saturation is reached at 12 participants (Braun and Clarke, 2013; Fugard and Potts, 2015; Guest et al., 2006).

2
https://www.sona-systems.com/

3
https://www.qualtrics.com/

4 The study included several quantitative measures, including assessments of individual differences: Questionnaire on cultural consciousness (van Klaveren et al., 2023), Bodily Sensation Maps (BSMs) (Nummenmaa et al., 2014; Schino et al., 2021; Nummenmaa and Hari, 2023), Generalized Anxiety Disorder (GAD-7) (Spitzer et al., 2006), Patient Health Questionnaire (PHQ-9) (Kroenke et al., 2001), Personality Attributes Questionnaire (PAQ-8) (Tibubos et al., 2022), and the Big Five Personality Test (BFPT) (Denissen et al., 2008). However, as these were not directly relevant to the present research objectives, we focused on emotional assessment using the Geneva Emotion Wheel (GEW).

5 Given the xy-coordinates of the point (*x,y*), with the circle center at (*x*_*centre*_ = 465, *y_*centre*_* = 316) and setting a minimum (*r*_*min*_ = 93) and a maximum (*r*_*max*_ = 239) radius, we calculated the normalized distance of a point from the center of the wheel, using the formula as shown. We took into account a minimum and maximum radius to avoid the parts where the buttons “None” and “Other” were displayed and the remaining frame outside of the wheel, respectively. The Euclidean distance between the point and the center is normalized by subtracting *r*_*min*_ and dividing by (*r*_*max*_–*r*_*min*_).

6
https://atlasti.com/es/atlas-ti-web

7
https://huggingface.co
