# Supplementary material for: Experiencing art together: integrating affect and semiosis
Source: Front Psychol. 2025 Apr 30;16:1544901. doi: 10.3389/fpsyg.2025.1544901 (PMC12075316; doi:10.3389/fpsyg.2025.1544901)
Supplement: Supplementary file 1 [file Supplementary_file_1.docx]

Supplementary Material

# Appendix A

**Supplementary Table 1A.** Prompts for the Dyadic Interaction.

| **Order** | **Prompt** | **Semiotic Strategy** |
| --- | --- | --- |
| 1 | Why did you decide to bring this artwork with you? What do you think about what your friend brought? | General |
| 2 | How does observing, touching, smelling, tasting, or listening to these artworks make you feel?  Do you find these artworks beautiful or not? and Why? | Perception |
| 3 | What can you do with these artworks? | Imagination |
| 4 | In what ways do you relate to these artworks? | Conceptualization |
| 6 | What purpose do these artworks fulfil by being made in this particular way? What would you like other people to know about these artworks? | Analysis |

**Supplementary Figure 1A.** Overview of the Objects Brought by Participants.


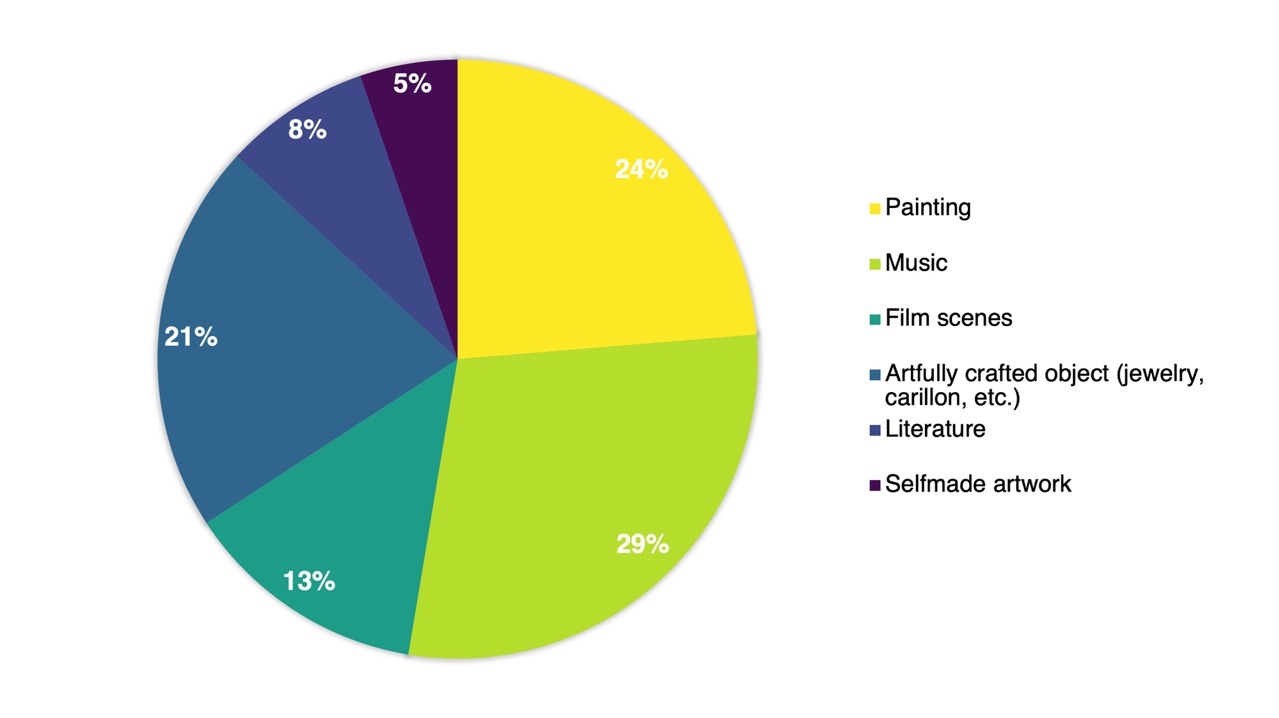


# Appendix B

There were two rooms used in the experiment, i.) experimental room - for the participants and the conduction of the experiment, ii.) control room

## Control Room

In the control room, the researchers monitored the participants, and controlled the display screen and audiovisual equipment. During the dyadic interactions, for audio and visual recordings, a microphone and video camera (Logitech BRIO) were used respectively and were checked from a computer in the control room. For recording and processing of audio and video two programs were used: AudioCapture (<https://github.com/labstreaminglayer/App-AudioCapture>) and SyncVideo (<https://github.com/markspan/VideoCapture>). We used LabRecorder (<https://github.com/labstreaminglayer/App-LabRecorder>) to save the audio and video streams to disk in XDF file format through LabStreamingLayer (LSL, <https://labstreaminglayer.org/>) open-source technology.

## Experimental Room

The experimental room was set up to recreate an informal, familiar atmosphere to foster a calm and safe environment between the participants during the conversation (see Figure 1B). The experimental room had two chairs, two small tables, two tablets (iPads), a long table, a display screen, audiovisual recording equipment and a laptop and/or headphones (if necessary). This set-up facilitated a more comfortable and naturalistic setting for participants. During art appreciation, tablets and headphones were provided by the researchers for art objects with audio/visual elements. For the questionnaires, tablets were provided by the researchers.

# Appendix C

This codebook serves as a manual developed to help coders identify strategies employed by the participants in their conversations about experiences of meaningful art objects. The goal of the coding procedure is to code a conversation between two participants and its content on underlying strategies employed by the participants.

## Process

Quotations are made in ATLAS.ti Web (<https://atlasti.com/es/atlas-ti-web>) from each specific utterance that needs coding.

The following coding levels have been used for the analysis:

Participant Number: Each participant has a specific number assigned to them, either: (a) Odd number; and (b) Even number. We coded each quotation of the transcription as Odd or Even according to the Participant number. To facilitate the pre-processing and analysis in R, we advise to adopt a coherent format among the transcripts and have each of these quotations starting with ‘P0’.

Strategy of sense-making: (a) Perception; (b) Imagination; (c) Conceptualization; (d) Analysis. A thorough description of the strategies, along with examples and keywords is provided in the coding scheme illustrated in Table 1 of this Appendix.

Utterances that do not fall under any coding level have been excluded from the analysis. These are sentences that portray no clear use of any strategy employed. Examples could be: A participant asking the other participant what he/she thinks; telling the other participant that the judgement was right; or rephrasing the prompt. On top of the above mentioned, utterances in the conversation that are not directed at the objects and their mutual exploration (observations in the lab room, talking about the weather, mentioning that one needs to go to the toilet or is thirsty) do not constitute quotations to be analyzed.

### Guidelines for Qualitative Data Analysis

These guidelines aim for clarity and consistency in qualitative data analysis of strategies, ensuring reproducibility and accurate interpretation of findings.

1. Distinguish into types of emotions per strategy:
   1. Perception: Direct and instinctual emotional responses. That is, all immediate reactions and basic emotions (potentially high arousal ones).
   2. Imagination: Imagined emotions without tangible triggers. That is, complex and fictional or quasi-emotions (potentially low arousal)
   3. Conceptualization: Representing emotions linked to abstract concepts or connected to the emotional significance to memories.
   4. Analytical: Insight emotional responses. That is, all emotions that derive from analytical processes.
2. When talking about past events we can use either the Imagination or Conceptualization strategy. If the participant is…
   1. …Recalling past events, that will be coded as use of Imagination strategy;
   2. …Talking about the attaching value attributed to those events, that will be coded as Conceptualization strategy.

Additionally, the coding approach will adopt the following rules:

- Often it is the case that multiple strategies are used in one sentence or utterance. This is logical because the strategies build upon each other. Try to find the main message and consider coding this message.
- Stick to literal expressions so as to keep misinterpretation to a minimum.
- Avoid coding standalone filler responses.
- Code repetitions if in different contexts or revealing novelty about the experience/participant.
- Coders should not break the sentence and maintain the point of continuity of the participants’ conversation or train of thought.
- If co-occurring thoughts in the same train of thought are detected, coders should apply additional coding. On the other hand, code separately if the thoughts can be assigned to different trains of thought.

**Supplementary Table 1C.** Coding Scheme for Semiotic Strategies.

| **Strategy** | **Description** | **Keywords** | **Real-data Examples** |
| --- | --- | --- | --- |
| Perceptual | Utterances and sentences corresponding to the strategy perception are those who explain a perceptual undertaking. Acknowledgement or experience. These are the concrete utterances of seeing, smelling, hearing and touching something but also utterances that explain the experience of a feeling that something can give someone. By using the perceptual strategy, the participants focus on the sensory qualities of an object. This strategy helps to appreciate the immediate impact of the object and how it elicits emotional and sensory responses. Sometimes the perceived object, or part of the object is not present, but participants refer to it from memory, this is considered part of the perception strategy as well, and not imaginary. | Seeing  Smelling Hearing  Touching  Feeling  Recognizing  Experience  Observe  Notice Consider | P37: “I could honestly feel how my shoulders were going down a lot.”  P38: “And since you brought them here I can touch them and feel them and - it is very sensory, very calming.”  P31: “I think it was a bit heavy, like, not pleasant to hold, so cold.”  P27: “Just like color-wise, I think the colors just complement each other very well, and I like the kind of contrast of the skin tone with like for example the brightness of the hair”  P23: “And it felt really nice to touch as well because it's a type of a stone and it's kind of cold…”  P30: “It is so beautifully drawn; the art style is so beautiful”  P01: “You can clearly hear the bass line, and the middle voice, and the melody” |
| Imaginative | The strategy of imagination concerns active and concrete manipulations of perceptions before us and in memory. For instance, thinking about a future condition. Using an object in a different matter than presented. Theorizing about how the world must have looked in the past, or, coming up with different endings to a music piece. The strategy enables us to engage our imagination and connect with objects (of art) on a more profound and subjective level. It also relates to the ability to see how someone else feels or thinks about something, or empathy. If something looks like something else (without comparing), this is done by employing an imagination strategy. An imaginary concept is also imaginary. | Design  Fantasize  Play  Pretend  Shape Would  Make  Create  Construct  Invent  Imagine If | P06: “my artwork moves me in the sense that it makes me want to find out, like to explore the world.”  P08: “I don’t know how to say, it makes you think about a faraway land.”  P25: “It’s so interesting to imagine that maybe somebody on the other side of the world has another Lego...”  P23: “And every time I read it, I read it in her voice... (laughs) I am also listening to it from her point of view”  P38: “My mother collected, uhm, like buttons. And this kind of reminds me of my grandmother’s collection, because feel like a huge bond with this” |
| Conceptual | Employing a conceptual strategy involves merging several concrete memories into abstract categories and underlying meaning. Judging, naming, classifying and labelling are all part of conceptualization. Conceptualization also involves extracting the underlying meaning of something. Many objects and artworks are created with specific concepts, ideas or cultural contexts in mind. The conceptual strategy allows us to understand underlying concepts, ideologies and cultural references involved in the artwork. As well as the maker’s intentions. Agreeing to something is always conceptual. Observations of abstract feelings like nostalgia and awe are considered part of conceptual strategies as well. | Judging  Naming  Classifying  Labelling  Represent  Belonging  To debate  To pronounce  To tell  Symbolize  To relate Nostalgic | P28: “I don’t know I kind of like that a little bit more than impressionistic view to paintings.”  P19: “I think, when - I’m a musician, personally, like music is very important to me”  P37: “I am impressed this is like a well-known symbol of peace and it’s something you brought, it’s universal, and I’m just like, wow that’s so powerful”  P11: “I mean, it’s my favorite singer and I think the song is really powerful and conveys a simple but significant meaning. And that is important to me”  P20: “It was an important game franchise for me too, so it was really cool”  P18: “TV series are less likely to be seen as art, while it is just - if you really just think about it- it’s just someone’s artwork” |
| Analytical | The analytical strategy involves abstract perception. What are the underlying structures of what we experience? What are the rules governing these structures? And what do the different parts of objects tell us about the world?  The analytical strategy helps us to deconstruct and analyze the formal and structural element of the object or artwork. It involves exploring composition, visual elements, artistic techniques and relationships between different elements within the work. We can extract artistic choices and the overall organization of an object.  Using arguments to support why something is made the way it was made is analytical as well. | Exploring Comparing Making connections Testing Made | P37: “The dove of peace is very simple, easy to reproduce and it was made this way. [...] I mean you can tell by the fact that it’s an international symbol of peace and everyone understands what it is about when they look at it, so simplicity and widespread”  P10:“Its purpose was probably capitalistic, because it was mass produced, but you can tell it was also made to be beautiful and sometimes all an artwork aims to be is beautiful”  P31: “You can find many little things in the drawing. Like some people don’t look at the drawing and they don’t look very long. And if you do you will find more things. So it’s supposed to make you do that”  P24: “And also like, for the little mistakes that she'd made she would add like a few, letters or words, and you can see that it’s just an innocent mistake, right, she’s adding more to it, scratching a few things out [...] You can see the thorough process and... Uhm... I guess the purpose of it, (it) was just more personal that way, because sometimes when it’s digital, ‘cause she could have done it digitally too, right?”  P18: “It is clearly a place that exists and you cannot change that, it is that place, but he has still made it very much his own, which I like. Just say, (with) those colors and such” |

# Appendix D

**Supplementary Table 1D.** Overview Table of Comparisons.

| **Comparison** | **Sentiment** | **Difference** | **p-value** | **p_BH** | **Direction** |
| --- | --- | --- | --- | --- | --- |
| Perception  vs  Imagination | Sadness | -0.053 | .706 | .706 |  |
|  | Love | 0.14 | .324 | .432 |  |
|  | Nostalgia | 0.212 | .024 | .085 | Perception > Imagination |
|  | Compassion | -0.089 | .498 | .569 |  |
|  | Joy | 0.044 | **.032** | .085 | Perception > Imagination |
|  | Enjoyment | 0.044 | **.032** | .085 | Perception > Imagination |
|  | Awe | -0.056 | .286 | .432 |  |
|  | Interest | -0.056 | .286 | .432 |  |
| Perception  vs  Conceptualization | Sadness | -0.202 | .142 | .379 |  |
|  | Love | 0.124 | .326 | .652 |  |
|  | Nostalgia | 0.015 | .878 | .878 |  |
|  | Compassion | -0.057 | .658 | .752 |  |
|  | Joy | 0.053 | **.008** | **.032** | Perception > Conceptualization |
|  | Enjoyment | 0.053 | **.008** | **.032** | Perception > Conceptualization |
|  | Awe | 0.023 | .626 | .752 |  |
|  | Interest | 0.023 | .626 | .752 |  |
| Perception  vs  Analysis | Sadness | 0.117 | .549 | .810 |  |
|  | Love | 0.039 | .863 | .863 |  |
|  | Nostalgia | -0.198 | .133 | .266 |  |
|  | Compassion | 0.426 | **.037** | .128 | Perception > Analysis |
|  | Joy | 0.069 | **.048** | .128 | Perception > Analysis |
|  | Enjoyment | 0.069 | **.048** | .128 | Perception > Analysis |
|  | Awe | -0.025 | .709 | .810 |  |
|  | Interest | -0.025 | .709 | .810 |  |
| Imagination  vs  Conceptualization | Sadness | -0.149 | .306 | .612 |  |
|  | Love | -0.016 | .916 | .916 |  |
|  | Nostalgia | -0.197 | **.026** | .208 | Imagination < Conceptualization |
|  | Compassion | 0.033 | .752 | .859 |  |
|  | Joy | 0.009 | .654 | .859 |  |
|  | Enjoyment | 0.009 | .654 | .859 |  |
|  | Awe | 0.079 | .104 | .277 |  |
|  | Interest | 0.079 | .104 | .277 |  |
| Imagination  vs  Analysis | Sadness | 0.17 | .436 | .661 |  |
|  | Love | -0.1 | .582 | .661 |  |
|  | Nostalgia | -0.41 | **.002** | **.016** | Imagination < Analysis |
|  | Compassion | 0.516 | **.010** | **.041** | Imagination > Analysis |
|  | Joy | 0.026 | .426 | .661 |  |
|  | Enjoyment | 0.026 | .426 | .661 |  |
|  | Awe | 0.031 | .661 | .661 |  |
|  | Interest | 0.031 | .661 | .661 |  |
| Conceptualization vs  Analysis | Sadness | 0.318 | .037 | .148 | Conceptualization > Analysis |
|  | Love | -0.084 | .666 | .666 |  |
|  | Nostalgia | -0.213 | .078 | .208 |  |
|  | Compassion | 0.483 | **.020** | .148 | Conceptualization > Analysis |
|  | Joy | 0.017 | .600 | .666 |  |
|  | Enjoyment | 0.017 | .600 | .666 |  |
|  | Awe | -0.048 | .495 | .666 |  |
|  | Interest | -0.048 | .495 | 0.666 |  |

**Supplementary Table 2D.** Overview of Exploratory Graph Analysis (EGA) Results and Bootstrap.

The present analysis used the GLASSO model (Graphical Least Absolute Shrinkage and Selection Operator, Friedman, Hastie, & Tibshirani, 2008), a common network estimation method that estimates a Gaussian Graphical Model (GGM) by calculating partial correlations between nodes. Furthermore, the Walktrap algorithm was applied to detect communities in the network, with random walks iterating over neighboring edges and prioritizing paths with larger edge weights (partial correlations). Additionally, a sparsity parameter, Lambda (or λ, n = 100, ratio = 0.1), was used to control network density. The Total Entropy Fit Index (TEFI) was computed to assess the fit of the dimensionality. Concerning the bootstrap procedure, we used a parametric one that generated (500) replicate samples based on the empirical partial correlation matrix. For further details on the methodology, consult (Christensen & Golino, 2021).

|  |  |  | **Perception** | **Imagination** | **Conceptualization** | **Analysis** |
| --- | --- | --- | --- | --- | --- | --- |
| **EGA** | *Number of Communities* | | 4 | 3 | 4 | 3 |
|  | *Number of Nodes* | | 8 | 8 | 8 | 8 |
|  | *Number of Edges* | | 18 | 8 | 12 | 8 |
|  | *Edge Density* | | 0.643 | 0.286 | 0.429 | 0.286 |
|  | *TEFI* | | -2.527 | -3.672 | -3.093 | -1.89 |
|  | *Lambda* | | 0.082 | 0.117 | 0.076 | 0.136 |
|  | *Non-Zero Edge Weights* | *M* | 0.085 | 0.142 | 0.142 | 0.173 |
|  |  | *SD* | 0.178 | 0.165 | 0.165 | 0.156 |
|  |  | *Min* | -0.091 | -0.072 | -0.072 | -0.052 |
|  |  | *Max* | 0.626 | 0.462 | 0.462 | 0.417 |
| **Bootstrap EGA** | *Median dimensions* | | 3 | 3 | 3 | 3 |
|  | *95% CI* | | 1.83, 4.17 | 2.45, 3.55 | 1.58, 4.42 | 1.71, 4.29 |
|  | *Structural Consistency* | *1* | 0.688 | 0.772 | 1 | 0.982 |
|  |  | *2* | 0.854 | 0.530 | 0.676 | 0.974 |
|  |  | *3* | 0.952 | 0.946 | 0.932 | 0.592 |
|  |  | *4* | 0.620 | – | 0.402 | 0.09 |
|  | *Proportion Replicated in Dimensions* | *Sadness* | 0.668 | 0.592 | 1 | NA |
|  |  | *Love* | 0.854 | 0.966 | 0.676 | 0.972 |
|  |  | *Joy* | 0.952 | 1 | 0.932 | 0.98 |
|  |  | *Awe* | 0.608 | 0.946 | 0.402 | 0.6 |
|  |  | *Nostalgia* | 0.666 | 0.444 | 0.676 | 0.992 |
|  |  | *Compassion* | 0.854 | 0.968 | 1 | NA |
|  |  | *Interest* | 0.608 | 0.946 | 0.402 | 0.592 |
|  |  | *Enjoyment* | 0.952 | 1 | 0.932 | 0.976 |

**Supplementary Table 3D.** Overview of Multinomial Model.

multinom(formula = quadrant ~ Semiotic_strategy * (joy + interest + love + nostalgia + awe) + (1 | Participant_id).

| **Response** | **Predictors** | **Log-Odds** | ***SE*** | ***t*** | ***p*** | **95% CI** |
| --- | --- | --- | --- | --- | --- | --- |
| **Quadrant (neg_lowAr)** | (Intercept) | 0.96 | 0.1 | 9.68 | **< .001** | [0.77, 1.16] |
|  | Analysis | 27.68 | 27.06 | 1.02 | .306 | [-25.37, 80.72] |
|  | Conceptualization | 0.71 | 0.48 | 1.49 | .136 | [-0.22, 1.65] |
|  | Imagination | -0.29 | 0.28 | -1.02 | .308 | [-0.84, 0.27] |
|  | joy | 0.37 | 0.21 | 1.74 | .081 | [-0.05, 0.79] |
|  | interest | -0.26 | 0.18 | -1.45 | .148 | [-0.61, 0.09] |
|  | love | -0.01 | 0.17 | -0.06 | .956 | [-0.35, 0.33] |
|  | nostalgia | -0.14 | 0.18 | -0.77 | .443 | [-0.50, 0.22] |
|  | awe | -0.16 | 0.16 | -0.98 | .328 | [-0.47, 0.16] |
|  | 1 \| Participant | 0.96 | 0.1 | 9.68 | **< .001** | [0.77, 1.16] |
|  | Analysis × joy | -1.42 | 0.61 | -2.33 | **.020** | [-2.61, -0.22] |
|  | Conceptualization × joy | -0.57 | 0.29 | -1.97 | **.049** | [-1.14, -0.00] |
|  | Imagination × joy | -0.25 | 0.3 | -0.84 | .401 | [-0.85, 0.34] |
|  | Analysis × interest | -0.35 | 0.51 | -0.69 | .491 | [-1.35, 0.65] |
|  | Conceptualization × interest | 0.66 | 0.36 | 1.81 | .070 | [-0.05, 1.37] |
|  | Imagination × interest | 0.34 | 0.29 | 1.18 | .236 | [-0.22, 0.91] |
|  | Analysis × love | 17.54 | 26.44 | 0.66 | .507 | [-34.28, 69.37] |
|  | Conceptualization × love | 0.04 | 0.28 | 0.16 | .875 | [-0.50, 0.58] |
|  | Imagination × love | -0.07 | 0.29 | -0.25 | .800 | [-0.65, 0.50] |
|  | Analysis × nostalgia | -0.58 | 0.71 | -0.81 | .416 | [-1.97, 0.81] |
|  | Conceptualization × nostalgia | -0.04 | 0.27 | -0.16 | .875 | [-0.57, 0.48] |
|  | Imagination × nostalgia | -0.07 | 0.25 | -0.29 | .772 | [-0.57, 0.42] |
|  | Analysis × awe | 45.13 | 52.44 | 0.86 | .389 | [-57.65, 147.92] |
|  | Conceptualization × awe | 1.12 | 0.98 | 1.14 | .255 | [-0.81, 3.05] |
|  | Imagination × awe | -0.05 | 0.24 | -0.21 | .836 | [-0.52, 0.42] |
| **Quadrant (pos_highAr)** | (Intercept) | 1.34 | 0.1 | 13.9 | **.001** | [1.15, 1.53] |
|  | Analysis | 27.63 | 27.06 | 1.02 | .307 | [-25.41, 80.68] |
|  | Conceptualization | 0.75 | 0.47 | 1.59 | .112 | [-0.18, 1.67] |
|  | Imagination | -0.21 | 0.27 | -0.76 | .445 | [-0.74, 0.33] |
|  | joy | 0.47 | 0.21 | 2.28 | **.022** | [0.07, 0.88] |
|  | interest | -0.29 | 0.17 | -1.68 | .092 | [-0.62, 0.05] |
|  | love | -0.06 | 0.17 | -0.35 | .726 | [-0.39, 0.27] |
|  | nostalgia | 0.06 | 0.17 | 0.36 | .718 | [-0.28, 0.41] |
|  | awe | 0.03 | 0.15 | 0.18 | .855 | [-0.27, 0.32] |
|  | 1 \| Participant | 1.34 | 0.1 | 13.9 | **.001** | [1.15, 1.53] |
|  | Analysis × joy | -1.23 | 0.6 | -2.05 | **.041** | [-2.40, -0.05] |
|  | Conceptualization × joy | -0.83 | 0.28 | -2.94 | **.003** | [-1.38, -0.28] |
|  | Imagination × joy | -0.14 | 0.29 | -0.47 | .638 | [-0.71, 0.43] |
|  | Analysis × interest | -0.17 | 0.5 | -0.34 | .733 | [-1.14, 0.80] |
|  | Conceptualization × interest | 0.83 | 0.35 | 2.35 | **.019** | [0.14, 1.53] |
|  | Imagination × interest | 0.48 | 0.27 | 1.75 | .079 | [-0.06, 1.02] |
|  | Analysis × love | 17.58 | 26.44 | 0.66 | .506 | [-34.24, 69.41] |
|  | Conceptualization × love | 0.12 | 0.27 | 0.45 | .651 | [-0.40, 0.64] |
|  | Imagination × love | 0.08 | 0.27 | 0.31 | .758 | [-0.45, 0.62] |
|  | Analysis × nostalgia | -0.68 | 0.7 | -0.97 | .330 | [-2.05, 0.69] |
|  | Conceptualization × nostalgia | -0.11 | 0.26 | -0.41 | .680 | [-0.61, 0.40] |
|  | Imagination × nostalgia | -0.14 | 0.24 | -0.58 | .559 | [-0.62, 0.33] |
|  | Analysis × awe | 45.03 | 52.44 | 0.86 | .391 | [-57.75, 147.81] |
|  | Conceptualization × awe | 1.05 | 0.98 | 1.07 | .286 | [-0.88, 2.97] |
|  | Imagination × awe | -0.18 | 0.22 | -0.79 | .427 | [-0.61, 0.26] |
| **Quadrant (pos_lowAr)** | (Intercept) | 1.48 | 0.1 | 15.44 | **< .001** | [1.29, 1.67] |
|  | Analysis | 27.78 | 27.06 | 1.03 | .305 | [-25.26, 80.83] |
|  | Conceptualization | 0.72 | 0.47 | 1.54 | .124 | [-0.20, 1.65] |
|  | Imagination | -0.16 | 0.27 | -0.58 | .562 | [-0.68, 0.37] |
|  | joy | 0.44 | 0.21 | 2.12 | **.034** | [0.03, 0.84] |
|  | interest | -0.32 | 0.17 | -1.88 | .061 | [-0.64, 0.01] |
|  | love | 0.08 | 0.16 | 0.48 | .631 | [-0.24, 0.40] |
|  | nostalgia | 0.02 | 0.17 | 0.12 | .903 | [-0.32, 0.36] |
|  | awe | 0.05 | 0.15 | 0.3 | .763 | [-0.25, 0.34] |
|  | 1 \| Participant | 1.48 | 0.1 | 15.44 | **.001** | [1.29, 1.67] |
|  | Analysis × joy | -1.24 | 0.6 | -2.08 | **.038** | [-2.41, -0.07] |
|  | Conceptualization × joy | -0.76 | 0.28 | -2.7 | **.007** | [-1.30, -0.21] |
|  | Imagination × joy | -0.17 | 0.29 | -0.58 | .559 | [-0.73, 0.40] |
|  | Analysis × interest | -0.28 | 0.49 | -0.56 | .573 | [-1.24, 0.69] |
|  | Conceptualization × interest | 0.8 | 0.35 | 2.28 | **.023** | [0.11, 1.49] |
|  | Imagination × interest | 0.39 | 0.27 | 1.44 | .149 | [-0.14, 0.92] |
|  | Analysis × love | 17.42 | 26.44 | 0.66 | .510 | [-34.41, 69.24] |
|  | Conceptualization × love | 0.02 | 0.26 | 0.08 | .939 | [-0.50, 0.54] |
|  | Imagination × love | -0.05 | 0.27 | -0.19 | .850 | [-0.58, 0.48] |
|  | Analysis × nostalgia | -0.55 | 0.7 | -0.78 | .433 | [-1.91, 0.82] |
|  | Conceptualization × nostalgia | -0.13 | 0.26 | -0.5 | .620 | [-0.63, 0.37] |
|  | Imagination × nostalgia | -0.15 | 0.24 | -0.63 | .531 | [-0.62, 0.32] |
|  | Analysis × awe | 45.1 | 52.44 | 0.86 | .390 | [-57.68, 147.89] |
|  | Conceptualization × awe | 1.07 | 0.98 | 1.1 | .273 | [-0.85, 2.99] |
|  | Imagination × awe | -0.21 | 0.22 | -0.96 | .337 | [-0.64, 0.22] |
